# Involvement of Huntingtin in Development and Ciliary Beating Regulation of Larvae of the Sea Urchin, *Hemicentrotus pulcherrimus*

**DOI:** 10.3390/ijms22105116

**Published:** 2021-05-12

**Authors:** Hideki Katow, Tomoko Katow, Hiromi Yoshida, Masato Kiyomoto

**Affiliations:** 1 Institute of Development, Aging and Cancer, Tohoku University, Sendai 980-8575, Japan; cic-admin.idac@grp.tohoku.ac.jp (H.Y.); kiyomoto.masato@ocha.ac.jp (M.K.); 2 Research Center for Marine Biology, Tohoku University, Aomori 039-3501, Japan; h.tkatow@d6.dion.ne.jp; 3 Marine and Coastal Research Center, Ochanomizu University, Chiba 294-0301, Japan

**Keywords:** huntingtin, CBAS, cell proliferation, ciliary beating, larval swimming, sea urchin larva

## Abstract

The multiple functions of the wild type Huntington’s disease protein of the sea urchin *Hemicentrotus pulcherrimus* (Hp-Htt) have been examined using the anti-Hp-Htt antibody (Ab) raised against synthetic oligopeptides. According to immunoblotting, Hp-Htt was detected as a single band at around the 350 kDa region at the swimming blastula stage to the prism larva stage. From the 2-arm pluteus stage (2aPL), however, an additional smaller band at the 165 kDa region appeared. Immunohistochemically, Hp-Htt was detected in the nuclei and the nearby cytoplasm of the ectodermal cells from the swimming blastula stage, and the blastocoelar cells from the mid-gastrula stage. The Ab-positive signal was converged to the ciliary band-associated strand (CBAS). There, it was accompanied by several CBAS-marker proteins in the cytoplasm, such as glutamate decarboxylase. Application of *Hp-Htt morpholino* (*Hp-Htt-MO*) has resulted in shortened larval arms, accompanied by decreased 5-bromo-2-deoxyuridin (BrdU) incorporation by the ectodermal cells of the larval arms. *Hp-Htt-MO* also resulted in lowered ciliary beating activity, accompanied by a disordered swirling pattern formation around the body. These *Hp-Htt-MO*-induced deficiencies took place after the onset of CBAS system formation at the larval arms. Thus, Hp-Htt is involved in cell proliferation and the ciliary beating pattern regulation signaling system in pluteus larvae.

## 1. Introduction

Huntington’s disease (HD) is a major neurodegenerative disorder in humans, involving involuntary body movements and dementia in the later stages, which is caused by the mutation of a gene, *huntingtin* (*HTT*). In the mutant Htt, the N-terminal region that contains cytosine-adenine-guanine (CAG) repeats expand extensively and subjected to be numerous neuropathological studies, including its molecular mechanisms [1]. The wild-type Htt, on the other hand, appears to be essential for embryogenesis [2], such as ciliogenesis and neurogenesis in Xenopus [3]. It is also involved in intracellular trafficking for extracellular matrix construction [4] and axonal transportation in mammals and a marine snail Aplysia [5]. HTT is required for maintaining mobility and long-term survival in Drosophila [6], and is also involved in regulating cerebrospinal fluid flow in humans [7,8,9,10]. HTT is present in marine invertebrates as well, such as the jellyfish *Cyanea capillata* [11], the marine snail *Aplysia* *californica*, *Branchiostoma floridae* [12], and the ascidian *Ciona intestinalis* [13], as well as the basal deuterostomes such as sea urchins, *Heliocidaris erythrogramma* (He-Htt) [12,14], and *Strongylocentrotus purpuratus* (Sp-Htt) [11,15,16]. However, there seems to be no neurodegenerative disorders in these animals, nor have CAG repeats been found to be involved in the above marine invertebrates to date. In fact, based on an in situ hybridization study, He-HTT and Sp-HTT were found to not be expressed in the neural tissues, such as the radial nerve in juveniles, but rather at mesodermal origins, including the hydrocoel, radial canals, and ampulae [15,16]. Thus, the evolutionary preserved basal functions of wild type HTT, if there are any, remain unknown to date.

The recent advancement of genome projects such as the Echinobase (https://www.echinobase.org/entry/), (Access on: 3 March 2021) which includes the sea urchin *Strongylocentrotus purpuratus* and several other marine invertebrates, and one that is specialized to the sea urchin, the *Hemisentrotus pulcherrimus* Genome and Transcriptome database (HpBase: http://cell-innovation.nig.ac.jp/Hpul/) (Access on: 3 March 2021) which annotated the HTT of *H. pulcherrimus*, Hp-Htt, has enabled us to proceed with the evolutionary analysis of HTT functions.

Based on these sea urchin DNA databases and the following examinations, the present study aimed to specify the role of the Hp-Htt protein during early developmental stages, from the swimming blastula (sBL) stage to the 6-arm pluteus stage (6aPL). We started by (1) specifying the dynamic Hp-Htt expression pattern by immunoblotting. This was followed by (2) immunohistochemical expression pattern analysis of the protein, (3) functional analysis of Hp-Htt during embryogenesis by knocking down the protein expression using *Hp-Htt morpholino* (*Hp-Htt-MO)*, (4) assessment of morphogenetic abnormalities by focusing on the cell proliferation activity, and finally (5) swimming behavior analysis of the larvae with and without *Hp-Htt-MO* based on ciliary beating activity analysis.

## 2. Results

### 2.1. Molecular Modification of Hp-Htt during Early Development

**Figure 1:**
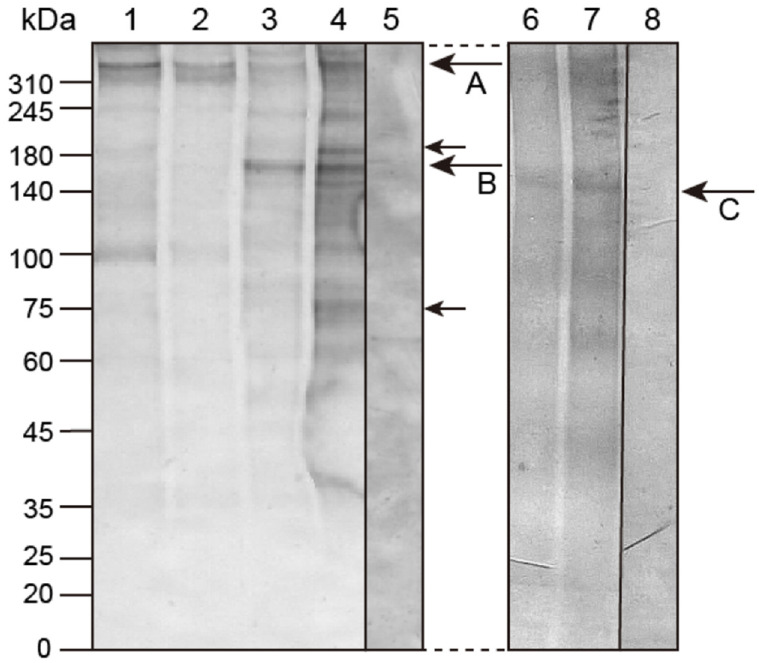
Hp-Htt expression during early development by immunoblotting. Lysates of embryos at the sBL stage (lane 1), the pL stage (lane 2), the 2aPL stage (lane 3), and the 4aPL stage (lane 4) were probed with anti-Hp-Htt Ab. For the negative control, 4aPL lysate was probed with a rabbit pre-immune serum (lane 5). An Ab-positive band was detected at around the 350 kDa region at the sBL and pL stages (arrow A). In 2aPL a new smaller band at the 165 kDa region was detected (Arrow B). In 4aPL, a further two new smaller bands (small arrows at 192 kDa and 75 kDa regions) appeared, in addition to the initial 165 kDa band. Anti-Hp-Tjp1-antibody detected the protein expression pattern of the pL stage (lane 6) and the 4aPL stage (lane 7). A single Ab-binding band was detected at the 150kDa region (Arrow C). The lysate was probed with a rabbit pre-immune serum (lane 8).

The anti-Hp-Htt antibody (Ab) detected a single band at around the 350 kDa region (Figure 1, arrow A) from the sBL stage to the prism larva (pL) stage. However, from the 2-arm pluteus (2aPL) stage to the 4aPL sage, the intensity of the immunoreaction of the initial major band declined, accompanied by the appearance of an additional new band of strong immunoreaction at the 165 kDa region (Figure 1, arrow B). In 4aPL, the middle section of the Hp-Htt that contained the antigen peptide sequence, further fragmented into two new smaller bands at 75 kDa and 192 kDa (Figure 1, smaller arrows). These immunoreactions were weaker than the initial two bands at the 350 kDa and 165 kDa regions. This may suggest ongoing Hp-Htt fragmentation during development, as will be discussed later.

To examine potential artificial fragmentation of Hp-Htt at and after the 2aPL, the other sea urchin protein Hp-Tjp1 (Tight junction protein 1) that is composed of 1402 amino acids and attains 154.4 kDa (HpBase: http://cell-innovation.nig.ac.jp/cgi-bin/Hpul_public/Hpul_annot_search_output.cgi) (Access on: 3 March 2021) and connects between embryonic ectodermal cells [17,18,19] was examined as a control for loading and lysate degradation.

At the pL stage, Tjp1 was detected at around 150 kDa region (Figure 1, lane 6, arrow C). At the 4aPL stage, the protein was detected at the same 150 kDa region (Figure 1, lane 7). The pre-immune serum, however, did not show any positive signal (Figure 1, lane 8), indicating fragmentation detected in Hp-Htt was not seen in Tjp1.

### 2.2. Spatiotemporal HP-Htt Expression Pattern

**Figure 2:**
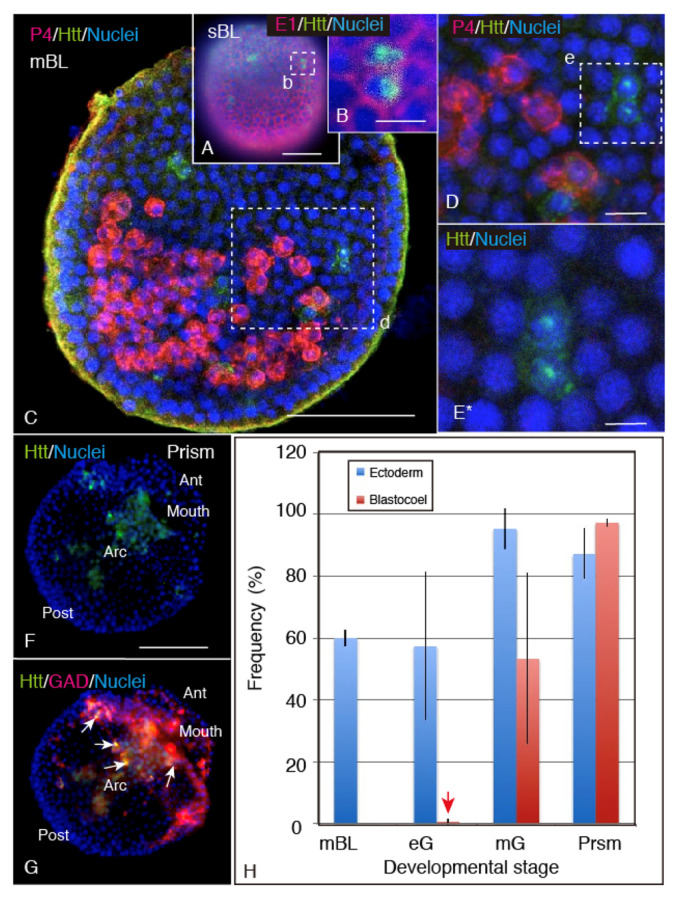
Fluorescent (**A**,**B**) and Confocal microscopic images (**C**–**G**) of Hp-Htt expressing cells and primary mesenchyme cells from the sBL stage to the pL stage. (**A**) Swimming blastula with an Hp-Htt-positive signal (green) in the Epith-2-positive ectoderm (red). (**B**) A higher magnification image of a region shown by the rectangle (b) in (**A**) shows an Hp-Htt-positive region in the nuclei of the ectoderm. (**C**) Confocal microscopy of mBL showing primary mesenchyme cells (PMC, red) that have invaginated into the blastocoel and the Hp-Htt-positive cells in the ectoderm (green). (**D**) Higher magnification image of the ectoderm indicated with a rectangle (d) in (**C**). (E*) Higher magnification of the cytoplasmic Hp-Htt-positive dots shown by a squire (e) in (**D**) shows the signal in the nuclei (blue) and the cytoplasm near the nucleus. Supplemental animation (Figure S2E*) shows a regional difference between the Hp-Htt-positive nuclear spot and the blurred Hp-Htt-positive area around the nuclei. (**F**) Ventral view of a late pL by fluorescent microscopy depicts the Hp-Htt-positive blastocoelar cells (green) around the archenteron (Arc) and the mouth opening (Mouth). Ant: Anterior, Post: Posterior. (**G**) A triple-stained image of (**F**) shows the Hp-Htt-positive signal (green) in the GAD-positive (red) cells (arrows). (**H**) Statistical analysis of spatiotemporal Hp-Htt-positive signal spreading from the ectodermal cells (blue columns) to the blastocoelar cells (red columns) during development from the mBL stage to the pL stage (Prsm). eG: early gastrula stage, mG; mid-gastrula stage. Red arrow; a tiny proportion of Hp-Htt-positive blastocoelar cells in eG. Scale bars = 50 µm (**A**,**C**,**F**), 15 µm (**B**), 10 µm (**D**), 5 µm (E*). (**D**) and (E*): Refer to Supplemental Figure S2E.

In the small number of ectodermal cells of sBL, Hp-Htt-positive signals were detected both in the cytoplasm and the nucleus (Figure 2A,B). In the mesenchyme blastulae (mBL), the cytoplasmic Hp-Htt-positive signal spread immediately beneath the cell surface, but no positive signal was detected among the primary mesenchyme cells (Figure 2C,D*). The nuclear Hp-Htt-positive signal of the ectodermal cells was seen as a strong spot (Figure 2D*,E*). In the late pL stage, the Hp-Htt-positive signal was mainly seen in the blastocoelar cells (Figure 2F), and most of these cells around the mouth opening expressed glutamic acid decarboxylase (GAD) as well (Figure 2G). Statistical examination of the Hp-Htt-positive cell distribution during the early development was consistent with the above respective immunohistochemical observations (Figure 2H). In mBL, the Hp-Htt-positive signal was exclusively localized in the larval ectoderm. In early gastrulae (eG), although a tiny proportion of blastocoelar cells expressed the Hp-Htt-positive signal (Figure 2H, red arrow at eG column), it was still significantly dominated by the ectodermal cells (*p*-value = 0.0146, *n* = 199). From the mid-gastrula stage, however, a substantial proportion of the mesenchyme cells expressed the Hp-Htt-positive signal. By the pL stage, the Hp-Htt-positive blastocoelar cells significantly exceeded the proportion of the Hp-Htt-positive signal in the ectoderm (*p*-value = 0.10061, *n* = 196).

**Figure 3:**
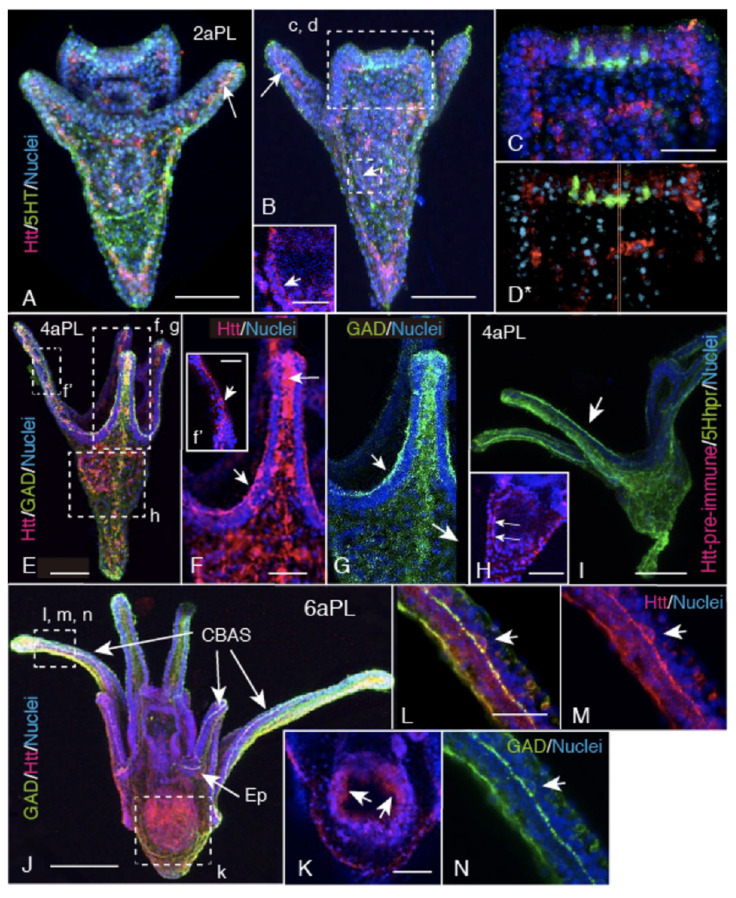
Confocal microscopy of triple- and double-stained plutei [Hp-Htt; red, serotonin (5HT); green, Serotonin receptor (5HThpr), green, GAD; green, and nuclei; blue)] showing the merged distribution of Hp-Htt-expressing cells and the CBAS during the pluteus stage. Hp-Htt-positive cells distribution was seen in the blastocoel on the basal surface of the ectoderm, and along the larval skeleton. (**A**) Ventral view of the triple stained 2aPL. Arrow: Hp-Htt-positive cells along the spicule. (**B**) Dorsal view. The Hp-Htt-positive cells being infiltrated into the ectoderm around the serotonergic apical ganglion. Arrow: Hp-Htt-positive cells were aligned along the arm spicule. (**C**) Higher magnification image of an apical ganglion region shown by a rectangle (c, d) in (**B**). Some of the Hp-Htt-positive cells near the apical ganglion shifted their position to the apical surface of the ectoderm. (D*) Animation of the same area as (**C**) shows spatial relations between Hp-Htt-positive cells and the apical ganglion. (**E**) In 4aPL, Hp-Htt-positive signals and GAD-positive signals were detected together in the CBAS, and in some of the blastocoelar cells. (**F**) Higher magnification of a rectangle (f, g) in (**E**) shows the CBAS (arrow). Long arrow: Hp-Htt-positive spicule tip area. Inset (f’): A higher magnification image of a rectangle (f’) in (**E**) showing a cytoplasmic Hp-Htt-positive signal along the axonal region and near the nuclei (arrow) in the CBAS. (**G**) The same area as (**F**) showing a GAD-positive signal (arrows). (**H**) A 3 µm thick optical section at around the middle of the stomach indicated by a rectangle (h) in (**E**) shows higher magnification of the Hp-Htt-positive stomach wall (arrows). (**I**) Four-arm pluteus triple stained with pre-immune rabbit serum (red) as a negative control of Hp-Htt-staining and anti-5HThpr antibody as a marker of the CBAS. (**J**) Six-arm pluteus triple stained for Hp-Htt, GAD, and DNA. These two signals of Hp-Htt and GAD are converged to the CBAS (arrows). The same signal conversion is seen at the CBAS of the epaulette (Ep). (**K**) Five µm thick optical cross-section around the middle of the stomach showing higher magnification of the stomach indicated by a rectangle (k) in (**J**). Arrows are the Hp-Htt-positive apical surface of the stomach. (**L**) Higher magnification image of a region shown by a rectangle (l, m, *n*) in (**J**) that depicts the perikaryon of the CBAS (arrow). (**M**) Higher magnification image of a region shown by a rectangle (l, m, *n*) in (**J**) of Htt-positive and nuclei image that depicts the perikaryon of the CBAS (arrow). (**N**) Higher magnification image of a region shown by a rectangle (l. m. *n*) in (**J**) double-stained for Hp-Htt and nuclei. Arrow; perikaryon. (**N**) The same area as (**M**) double-stained for GAD (green) and nuclei (blue). Scale bars = 50 µm (**A**,**B**,**E**,**H**,**K**), 25 µm (**C**,**F**), 75 µm (**I**), 150 µm (**J**), 20 µm (**L**), 10 µm (f’). (D*): Refer to Supplemental Figure S3D.

From the 2aPL stage, serotonin was intensively expressed at the apical ganglion and the Hp-Htt-positive signal was detected in the blastocoelar cells around the apical ganglion (Figure 3A–C), and in those in the larval arms along the spicule (Figure 3A,B,F, arrow). In the blastocoel, these Hp-Htt-positive cells were distributed on the basal region of the ectoderm (Figure 3A,B). At the apical ganglion region, both serotonin- and Hp-Htt-positive signals were detected at the respective region without overlapping each other (Figure 3C,D*). In 4aPL, the Hp-Htt-positive signal was detected in the GAD-expressing ciliary band-associated strand (CBAS) as one of the constituting proteins (Figure 3E,G). Although the GAD and Hp-Htt expressing regions in the axonal region of the CBAS were very close, their positive signals were not exactly overlapping in the cytoplasm (Figure 3F,G).

Hp-Htt was detected in the cytoplasm of the perikaryon but not in the nuclei of the CBAS (Figure 3f, arrow). By this developmental stage, Hp-Htt-expressing blastocoelar cells formed a large cellular network (Figure 3E,F). Toward the 6aPL stage, the Hp-Htt-positive signal was also detected at the apical surface of the stomach wall epithelium (Figure 3E,H,J,K). At the 6aPL stage, while the co-expression of GAD (or 5HThpr in Figure 3I) and Hp-Htt remained the same (Figure 3I,J), there was still a different distribution in the perikaryon of the CBAS. While Hp-Htt was expressed both at the perinuclear cytoplasm and the axonal region (Figure 3L,M), the GAD-positive signal was not detected in the perikaryon, but was at the axonal region (Figure 3L. Thus, in plutei, the Hp-Htt-positive signal was detected associated with cilia, such as at the CBAS and the digestive ducts, including the stomach wall cells and the esophagus. However, at the ectoderm of the apical tuft region, which extend long cilia, the positive signal was not detected (Figure 3A–C).

### 2.3. Hp-Htt Gene Knockdown by Its Morpholino Antisense Oligomers

In 4aPL, the present study detected Hp-Htt in the CBAS, along with GAD and 5HThpr. According to our previous study [19], this implies the involvement of Hp-Htt in ciliary beating activity. To verify this, *Hp-Htt-MO* was introduced into the embryos using Endo-Porter-PEG (PEG), according to the instructions of the manufacturer (GeneTools, LLC. Philomath, OR 97370 USA), and we examined its effects on CBAS formation and ciliary beating.

**Figure 4:**
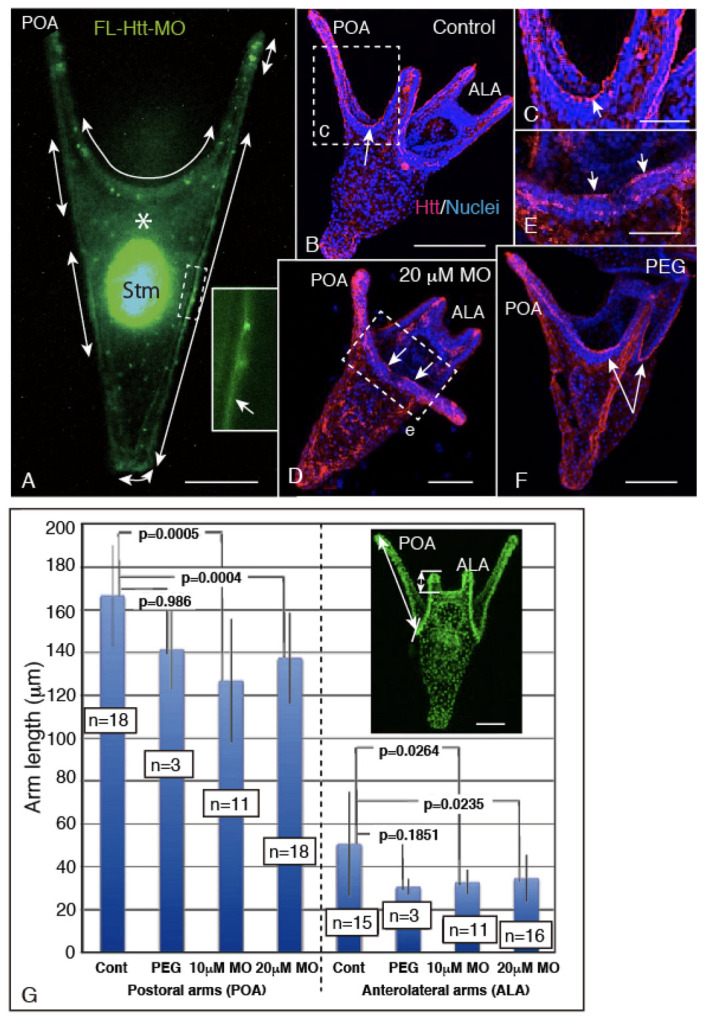
Shortened larval arm length in *Fluorescein-tagged Hp-Htt morpholino* (*FL-Htt-MO*)-treated 4aPL. (**A**) Fluorescence microscopy of *FL-Htt-MO*-positive areas (green) in the stomach (Stm), the ectoderm regions (double-headed arrows), and the blastocoelar space (asterisk). Inset: Higher magnification of green glow cytoplasmic region of the ectoderm (arrow) of the larval trunk, shown by a dotted-line rectangle. (**B**–**F**) Confocal microscopies double-stained for Htt (red) and nuclei (blue). (**B**) Control larva. Hp-Htt was detected at the CBAS (arrow). (**C**) Higher magnification image of a doted-line rectangle (c) in (**B**) shows CBAS by an arrow. (**D**) 20 µM *FL-Htt-MO*-treated larva. Larval arms are distinctively shorter than those in the control larva and CBAS was fragmented (arrows). (**E**) Higher magnification image of a dotted-line rectangle (e) in (**D**). The Hp-Htt-positive area shows a fragmented CBAS (arrows). (**F**) PEG-treated larva extends normal length arms with continuous Hp-Htt-positive signal in CBASs (arrows). (**G**) Statistical analysis of the arm length shown by columns with error bars depicting the shortened arm length in the *FL-Htt-MO*-treated larvae. The number of larvae examined is indicated in boxes in the middle of each column. *p* values were calculated between the two columns connected with solid lines. Inset: The dorsal view of a pseudo-green-colored DAPI-stained larva shows places that were subjected to measurement of the arm length with double-headed arrows. Scale bars = 50 µm (**A**,**G** inset), 100 µm (**B**), 25 µm (**C**,**E**), 75 µm (**D**,**F**).

First, the hatched pL were incubated with 20 µM fluorescein-tagged *Hp-Htt-MO* (*FL-Htt-MO*) during the following developmental period until the 4aPL stage. The fluoresceinated compound was seen as diffuse fluorescence throughout the cytosol (Gene Tools, https://www.gene-tools.com/morpholino_antisense_oligos) (Access on: 3 March 2021) of the ectoderm (Figure 4A, double-headed arrows) and in the blastocoelar space (Figure 4A, asterisk). A large *FL-Htt-MO*-positive area is a stomach and it was swallowed during mouth formation at around the late pL stage.

Aliquots of the 4aPL sample were subjected to whole-mount immunohistochemistry (WMIHC) analysis using anti-Hp-Htt Ab. While the control 4aPL formed the Hp-Htt-positive CBAS between two postoral arms (Figure 4B,C), the *FL-Htt-MO* larvae extended shorter arms, with fragmented Hp-Htt-positive signal at their CBAS (Figure 4D,E, arrows). Another negative control 4aPL that was introduced with PEG alone did not form such CBAS with fragmented Hp-Htt-positive signals (Figure 4F). The disturbed arm extension due to *FL-Htt-MO* was subjected to statistical analysis (Figure 4G). While PEG did not affect the arm elongation (*p* = 0.986), *FL-Htt-MO* significantly repressed the elongation of the postoral arms (*p* = 0.0005 for with 10 µM MO, *p* = 0.0004 with 20 µM MO). The anterolateral arms were also significantly affected both in two concentrations of MO (*p* = 0.0264 with 10 µm MO, *p* = 0.0235 with 20 µm MO).

### 2.4. FL-Htt-MO Formed Shortened Larval Arms and Showed Decreased Cell Proliferation

**Figure 5:**
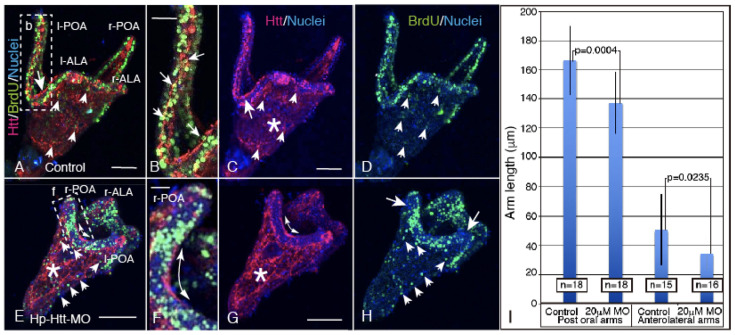
Hp-Htt (red)/BrdU (green)/nuclei (blue) triple stained image of 4aPL. Shortened larval arms due to *FL-Htt-MO* application occurred, accompanied by decreased BrdU-positive signals, particularly at the larval arm regions. (**A**) Dorsal view of the larva. Hp-Htt was detected at the CBAS (large arrow) and some of the blastocoelar cells (small arrows). (**B**) The highly magnified CBAS area shown by a rectangle (b) in (**A**). BrdU was detected also there (arrows). (**C**) Hp-Htt/DAPI double stained image of (**A**). Arrow; CBAS. Hp-Htt-positive blastocoelar cell network (asterisk). (**D**) At the ectoderm, a smaller number of BrdU-positive signals were detected in the blastocoelar cells (arrows) **(E**) Ventral view of the *FL-Htt-MO* treated larva. The number of BrdU-positive signals clearly decreased in the ectoderm, particularly at the tip of arms and in the fragmented CBAS (double-head arrow). (**F**) The highly magnified area shown by a rectangle (f) in (**E**). Shorter arm length is clear at the postoral arms (POA). (**G**) Fragmented CBAS (double-head arrow in **F** and **G**). The blastocoelar Hp-Htt-positive cell network (asterisk) is rather unaffected. (**H**) BrdU-positive signal image extracted from (**E**) clearly detecting the declined positive signal at the tip regions of the arms (arrows), while those in the blastocoel were not apparently decreased (small arrows). (**I**) Statistical analysis of average arm length comparison between control larvae and *FL-Htt-MO*-applied larvae with *p*-value. The larval number examined are shown in a box on each column. Scale bars = 50 µm (**A**,**C**,**E**,**G**). 25 µm (**B**,**F**).

To examine the mechanism of the above larval arm shortening, the potential involvement of decreased cell proliferation activity was assessed by immunohistochemically detecting BrdU incorporation and its locations. Shortly after hatching, at around the mBL stage, the embryos were incubated with *FL-Htt-MO* and then kept at the 37-hpf 4aPL stage for 3 h with BrdU. In control *FL-Htt-MO*-free larvae, BrdU-positive signals were concentrated at the circumoral ectoderm, which includes the larval arms, in four larvae of the total of five larvae examined (Figure 5A). These BrdU-positive signals were also detected, with a few at the CBAS (Figure 5B arrows,C,D), implying that the larval arm extension during the above period was carried out mainly by the circumoral ectodermal cells. In the *FL-Htt-MO*-treated larvae, however, the BrdU-positive signals apparently declined at the larval arm tips in four larvae of the total six that were examined (Figure 5E,H). At the fragmental Hp-Htt-positive signal regions of the CBAS region, BrdU-positive signals were not detected at all, which appears to be consistent with the following observation that fewer BrdU positive signals were seen during larval arm extension (Figure 5E,F,G, double-headed arrow, H). In the blastocoel, while fewer BrdU-positive cells were seen in the control 4aPL, no visible inhibition of the blastocoelar network formation by Htt-positive cells was seen in the *FL-Htt-MO* treated larvae (Figure 5C,E,G, asterisk).

### 2.5. Disruption of the Swirling Track Pattern Created by the Ciliary Beating Activity in Hp-Htt-MO Embryo and Plutei

The present immunohistochemical detection of Hp-Htt in the CBAS implied the involvement of the protein in ciliary beating activity. Swimming of sea urchin embryos depends on the ciliary beating on their body surface, accompanied by the swirling water current it generates, before the pluteus stage. At and after the 2aPL stage, the main body of the ciliary beating activity shifts from the earlier entire embryonic body surface to the ciliary band that is formed on the larval arms. To visualize the swirling water current pattern generated by the ciliary beating activity, the present study recorded the swirling track patterns using a marker of tiny particles. In this study, the algae *Chaetoceros gracilis*, whose cell size attains around 5~7 µm in diameter, was used [19,20].

**Figure 6:**
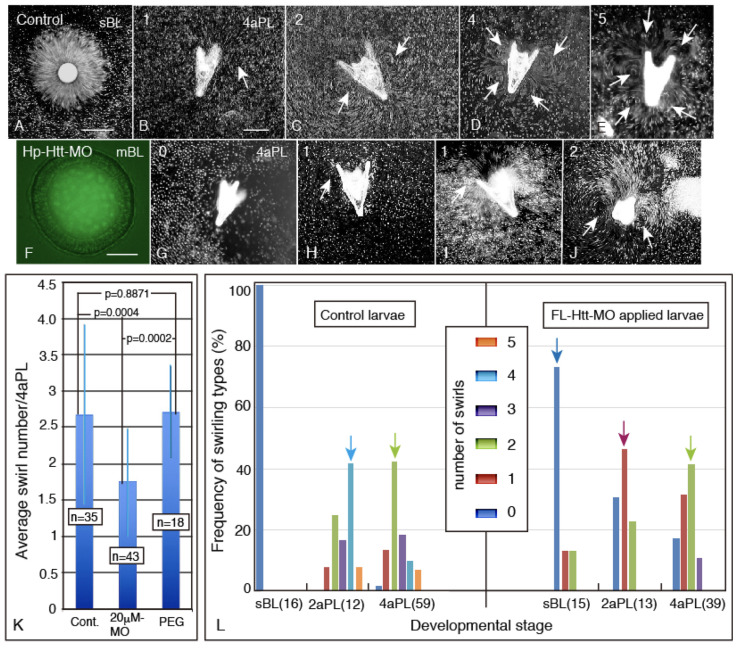
Increasing number and types of swirling patterns (arrows) during development. (**A**) A water current around the body surface with no swirling pattern of the control sBL. (**B**–**E**) Control 4aPLs with different numbers of swirling patterns. The number on the upper left corner is that of swirls. (**F**) Fluorescence microscopy of *FL-Htt-MO* in the blastocoel of mBL before fixation. (**G**) An *FL-Htt-MO*-treated 4aPL generated no water current around the body. (**H**–**J**) The swirling patterns of *FL-Htt-MO*-treated 4aPL. The upper left number shows that of swirls. The arrows point to the swirls. (**K**) The average number of swirlings of 4aPL of control (Cont), *FL-Htt-MO* (20 µM MO)-treated, and Endo-Porter PEG (PEG) alone. The numbers in boxes on each blue column indicate those of the samples. Thinner blue vertical bars on the top of each column show the standard deviations. *p* values are shown between two subjected columns connected with lines. (**L**) Frequency of the swirling pattern types, from sBL to 4aPL, are shown in the central box, with dark blue for zero swirls to five swirls in the orange column. The control larvae are on the left side and the *FL-Htt-MO*-treated larvae on the right side of the graph. The numbers in parentheses at the end of the developmental stage names show that of samples that were examined in that stage. The light blue arrow in the control group points to the major swirling pattern group in the control 2aPL. The green arrow shows the major swirling pattern group in the control 4aPL. The dark blue arrow at the top of the dark blue column of the *FL-Htt-MO*-treated groups shows the major swirling type of the sBL. The brown arrow at the top of the brown column shows the major swirling type of the 2aPL. The light green arrow at the top of the green column in the *FL-Htt-MO*-treated groups shows the major swirling type of the 4aPL. Scale bars = 100 µm (**A**), 50 µm (**B**), 30 µm (**F**).

The control swimming blastulae created a water current on their body surface, but with no particular swirling tracking pattern, only a radial current around the body (Figure 6A). The 4aPL, however, generated diverse patterns of swirls, from unilateral one swirl (Figure 6B), bilateral one pair of swirls (Figure 6C), bilateral four swirls (Figure 6D), and up to five swirls (Figure 6E). In *FL-Htt-MO*-treated embryos, soon after the insemination, diffuse fluorescence was visible in the blastocoel and the ectoderm. This was the *FL-Htt-MO* entrapped in the intercellular space during cleavage, and then entrapped finally in the blastocoelar space (Figure 6F). This was regarded as successful delivery of *FL-Htt-MO* to the cytoplasm by the present method (Gene Tools, LLC, https://www.gene-tools.com/content/getting-morpholinos-cultured-cells) (Access on: 3 March 2021). They swam slowly near the bottom of the 24-well plate, and occasionally up to the surface. At the 4aPL stage, the major proportion of *FL-Htt-MO*-introduced larvae generated either no swirl (Figure 6G), one swirl (Figure 6H,I), or two swirls as a major group (Figure 6J). Only a tiny proportion of larvae generated three swirls at most, as the smallest group, (Figure 6L, right columns) which was a considerable decline in proportion from the control group as the second major group (Figure 6L, left columns), as will be described in detail below.

Statistical examination using a larger number of larvae detected an average of 2.5 swirls in control larvae, while *FL-Htt-MO*-treated ones generated an average of 1.5 swirls; this was a statistically significant difference, *p* = 0.0004 (Figure 6K). Without *FL-Htt-MO*, with the additive PEG alone, the swirl number stayed at an average of 2.6; it did not show a decline (*p* = 0.8871) (Figure 6K). The swirl number increased and the patterns were diversified in control larvae during development from the sBL stage to the 4aPL stage. To verify the effects of *FL-Htt-MO*, the swirling generation patterns were examined in detail (Figure 6L). The control sBL largely generated no swirl (98%), but a very small proportion of them formed a single swirl (Figure 6L, left columns). The control 2aPL generated five types of swirling patterns, with the 4-swirls as the major group (Figure 6L, left columns, blue arrow). The control 4aPL generated up to five swirling types, with the 2-swirls as the major group (Figure 6L, left columns, green arrow). On the other hand, the *FL-Htt-MO*-treated sBL generated no swirls as the major group (about 80%) (Figure 6L, right column, dark blue column). The rest of the larvae generated a small proportion of one or two swirls, at 10% (Figure 6L, right columns). In the *FL-Htt-MO*-treated 2aPL, the single swirl type was the major group (Figure 6L, brown arrow in right columns), and the no-swirl group appeared, with a radical increase from none. In the 4aPL, while 1- to 2-swirl types were the major groups, the 2-swirls group declined noticeably from the control (Figure 6L, right columns, green arrow). Thus, *FL-Htt-MO* reduced the number of swirls and swirling types and the diversity in the larvae older than 2aPL. This indicated significant involvement of Hp-Htt in ciliary beating behavior for swirling pattern formation in larvae.

## 3. Discussion

### 3.1. Hp-Htt Expression Pattern during Development

Huntingtin is a large single molecule of around 348 kDa (HD-Human; P42858; [15,16,21,22,23]) to 350 kDa [24,25,26,27,28] in humans, and around 339 kDa in the sea urchin *Strongylocentrotus purpuratus* [Sp-Hunt (Sp-Htt); XP_030841199.1, XP_030848870.1]. The present immunoblotting of *H. plucherrimus* (Hp-Htt) detected a single band of around 350 kDa from the swimming blastula stage to the pL stage, indicating that the size is very close to those of above HD-human and Sp-Htt, but larger than that of *Ciona intestinalis* [15]. However, at the 2aPL stage, the Hp-Htt was split into two bands by creating a new smaller 165 kDa fragment, in addition to the initial around 350 kDa fragment. This may be related to the observation that the immune intensity of the initial single band decreased (a possible quantitative decrease of the initial 350 kDa molecule) from the 2aPL stage to the 4aPL stage (Figure 1). Moreover, two further smaller bands appeared from the 2aPL stage in relation to the beginning of the decreased relative immunointensity of the 350 kDa band region. Considering the results of previous immunoblotting studies conducted using multiple Abs against human HTT, with multiple splitting producing smaller bands [28], the present smaller bands might be the consequence of similar fragmentations of the initial 350 kDa Hp-Htt.

Furthermore, such fragmentation was seen more often in the nuclei than in the cytoplasm [29]. In this process, given the presence of the 10 peptides-leucine-rich nuclear export signal (NES), which includes seven conserved peptides of the SLARLPL sequence in the *N*-terminal of vertebrates, Htt is said to control subcellular localization [24]. Similar poly-peptides are present in Hp-Htt as ^129^SLGRLQL^135^ [HPU_11727. Hp-Hunt (Hpbase, http://cell-innovation.nig.ac.jp/Hpul/)] and in Sp-Htt [SPU_011485.1 (EchinoBase, http://legacy.echinobase.org/Echinobase/Search/SpSearch/viewAnnoGeneInfo.php?spu_id=SPU_011485)] (Access on: 3 March 2021) with the same constitution as ^104^SLGRLQL^111^.

Although the proteolytic mechanism and potential functional significance of the present Htt fragmentation during early development is yet to be examined, cleavage of wild-type Htt in normal development has been reported in humans as well [30]. It occurs by caspase-6 and -1 activity in both normal brain and Huntington’s disease brains. Thus, the cleavage itself is not regarded to be a disease-specific phenomenon, and the caspase cleavage of Htt can be a normal physiological event [28,31]. In healthy conditions, proteolysis in mice is limited and the C-terminal fragments generated to interact with the *N*-terminal fragments preventing toxicity of the C-terminal [29].

The present WMIHC detected Hp-Htt mainly in the nuclei, accompanied by several weak cytoplasmic signals in the ectoderm of mBL. During the later stages to the 6aPL stage, Hp-Htt was detected in the cytoplasm of blastocoelar cells (Figure 3F) and the perikaryon, including the axonal regions of the CBAS of 4aPL (Figure 3f) and 6aPL (Figure 3L,M). Although the above morphological developmental stages are when Hp-Htt fragmentation was detected by immunoblotting, whether the NES signal is involved in this cytoplasmic localization of Hp-Htt remains to be examined. To date, however, at least in Drosophila-Htt, no such correlation has been reported yet [2].

CBAS formation starts from the 2aPL stage, accompanied by the blastocoel-to-larval body surface egression of the GAD-expressing blastocoelar cells between the oral ectoderm and the ciliary band [18,22,23,24,25]. On the other hand, the other GAD/Hp-Htt-expressing cells left in the blastocoel form the large blastocoelar networks (Figure 3E–G, Figure 5C,G). These GAD-expressing cells also express Hp-Htt both in the CBAS and the blastocoel. They are located spatially very close in the cytoplasm, as shown in this study (Figure 3E–G). Such spatial closeness between these molecules in the same cell cytoplasm suggests the occurrence of a functional relationship that regulates the cytoplasmic GABA level, as has been reported in the caudate/putamen of the neostriatum human forebrain [4]. However, a decisive interaction mechanism is yet to be elucidated. The present observation also detected the expression of Hp-Htt near the ciliated ectodermal cells. These cilia, including those in the stomach, are characterized by active beating. At the apical tuft region near the serotonergic apical ganglion, on the other hand, long cilia do not beat as actively as the shorter cilia in the other regions [20]. However, we did not detect Hp-Htt at or around the basal body of the cilia of the ciliary band; rather, Hp-Htt was located in the CBAS, which contains several signaling proteins, such as encephalopsin [19,24], synaptophysin [19,22,25], and 5HThpr [19,32]. Hp-Htt is also located in stomach wall cells (Figure 3B inset, H). Thus, Hp-Htt could be involved in the ciliary beating regulation mechanism, implicating that the CBAS is a signal transmission system other than the known sea urchin nervous system that expresses 1E11 [23] which, however, does not express Hp-Htt.

### 3.2. Swirling Patterns

Sea urchin embryos swim actively soon after their hatching through active ciliary beating that takes place on the body surface, and then at the ciliary band during the pluteus stages. Then, they settle down on a substrate, such as rocks or seaweed, for metamorphosis. These cilia are accompanied by dopamine- and dopamine receptor D1 (Hp-DRD1)-positive basal granules from the 8-hpf blastula stage to the developmental stage that is immediately before the onset of metamorphosis [33]. During these larval stages, the ciliary band emerges, followed by CBAS formation on the oral ectoderm side. This takes place following the egression of the blastocoelar mesenchyme cells to the larval body surface. These blastocoelar cells express several signal transmission-related proteins, such as GAD, 5HThpr, encephalopsin, and synaptophysin as stated above, in addition to Hp-Htt. As has been shown in this study, the larval swimming response to *FL-Htt-MO* was clearly different after the larval arm formation.

The inhibitory effect of *FL-Htt-MO* on the body surface swirl generation was seen only after the CBAS formation in the larvae and was accompanied by the Hp-Htt-positive signal declining and fragmentation of Hp-Htt-positive signal in the CBAS. This implies Hp-Htt’s deep involvement in the ciliary beating activity for larval swimming. The water current generation by ciliary beating also has been reported in the cerebrospinal fluid (CSF) flow in the vertebrate brain [34,35,36,37,38,39,40], and involvement of Htt has been reported through ciliogenesis [23,36,41]. However, how Htt is involved in ciliary beating regulation in CSF flow, other than ciliogenesis, remains unclear [40].

In aquatic invertebrates, the ciliary beating activity is regulated by ciliomoter neurons [42,43], such as acetylcholinergic and serotonergic neurons in Platynereis larvae [44], and dopaminergic and serotonergic neurons in sea urchin larvae [19,33]. These neurotransmitter mechanisms, such as the GABAA receptor for GABA transmission, involve Huntingtin [23,45]. In sea urchin larvae, since GABA [19,22,23] and GABAA receptor are present in the ciliary band [23] and GAD is present in the CBAS of sea urchin larva [19], the histological Hp-Htt location may indicate the presence of its regulatory function in ciliary beating through the neurotransmission process.

### 3.3. Htt in Morphogenesis

Htt is reported to be widely expressed during development and thus is suggested to interact with a large number of effector proteins [26]. Among them, wild-type Htt is reported to protect CNS cells from a variety of apoptotic stimuli by caspase-9 activation and also is shown to be involved in ciliogenesis [46] and is regarded to be essential for embryonic development in mice [23]. Although there can still be some controversy on the involvement of Htt in morphogenesis, according to Htt knockout experiments in zebrafish and rodents, postnatal development, such as body size, seems to involve wild-type Htt [47].

In the present study, *FL-Htt-MO* application to the plutei resulted in shorter larval arms being associated with decreased BrdU incorporation, particularly around the arm tip region. This indicated decreased cell proliferation there due to *HL-Htt-MO*. Htt involvement in mitotic spindle orientation in Drosophila [38] and mammalian cells [27,38] has also been reported. Furthermore, these shortened larval arms were accompanied by shortened spicules. The presence of Hp-Htt-positive blastocoelar cells around the spicules (Figure 3F) may not be in the primary mesenchyme cells, as has been shown in this study (Figure 2C). Thus, these Hp-Htt-positive cells may have a role in spicule pattern formation.

## 4. Materials and Methods

### 4.1. Animal Preparation

Sea urchins, *H*. *pulcherrimus*, were collected in the vicinity of the Research Center for Marine Biology, Tohoku University, Japan, or the Marine and Coastal Research Center, Ochanomizu University, Japan. The gametes were collected by intracoelomic injection of 0.5 M KCl, inseminated in vitro, and incubated at 17–18 °C until the 6aPL stage in filtered sea water (FSW), as has been described before [32]. Some of the gametes were preserved after shedding in antibiotic diluted filtered sea water [33], and utilized as stated above.

### 4.2. Raising Antibody 

**Figure 7:**
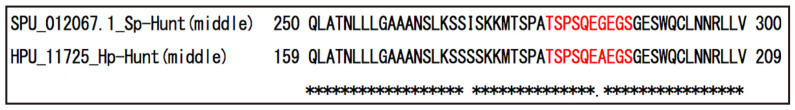
The antigen peptide sequence (red letters) and its location in the Sp-Htt protein (SPU_012067.1_Sp-Hunt: https://www.echinobase.org/entry/; Hp-Htt (HPU_11725_Hp-Hunt: HpBase: http://cell-innovation.nig.ac.jp/Hpul/ [48] (Access on 5 May 2021).The respective sequence is located in the middle section of the protein, and is very similar.

The epitope of anti-Huntingtin Ab of Sp-Htt was NH2-C+^274^TSPATSPQEGEGS^287^-COOH in the amino acid sequence (SPU_012067.1) registered in EchinoBase (http://legacy.echinobase.org/Echinobase/AboutEB) (Access on: 3 March 2021), and was chosen based on GenScript’s Optimum Antigen design tool (https://www.genscript.com/antigen-design.html) (Access on: 3 March 2021) analysis. The sequence also was reported in the earlier report on Sp-Htt [14]. The homologous amino acid sequence is also present in Hp-Htt [Hp-Hunt (middle), HPU_11725], as well as with one peptide difference at ^283^G in *S. purpuratus* and ^141^A in *H. pulcherrimus* (Figure 7).

The antigen, NH_2_-CTSPATSPQEGEGS-COOH, was synthesized and utilized for raising antibody in rabbits by Eurofins Genomics K.K (Ota-ku, Tokyo, Japan 143-0003). Before inoculation, blood serum was collected from the rabbits and used as a pre-immune serum.

### 4.3. Immunoblotting

The embryos at the sBL stage, the pL stage, the 2aPL stage and the 4aPL stage, were centrifuged to collect packed 10 µL precipitations and were dissolved immediately in cold 500 mL RIPA Buffer (Fujifilm Wako Pure Chemical Co. Osaka, Japan), incubated for 5 min on ice, and stirred at 2000 xg for 15 min. The resultant supernatants were aliquoted at 50 µL in 0.2 mL EU PCR tubes (Nippon Genetics Co., Ltd., Tokyo, Japan) and kept at –20 °C until use.

These samples were separated by SDS-poly acrylamide gel electrophoresis using Multigel II Mini 2/15 (2~15%) gradient gels (Cosmo Bio Co. Ltd., Tokyo, Japan), and blotted to Whatman Protoran BA 85 Nitrocellulose filters (GE Healthcare Japan Co., Hino, Tokyo, Japan). The blotted membranes were blocked with 5% Skim Milk diluted in 0.1 M TBST, probed with anti-Hp-Htt Ab, anti-Hp-Tjp1 Ab [18] or pre-immune serum diluted in 0.1M TBST with 5% Skim Milk diluted at 1:3000, and incubated on a rocking deck overnight at 4 °C. The Ab was detected by incubating the blotting membrane for 2 h at room temperature in alkaline phosphatase-conjugated anti-rabbit IgG (Fc) (Promega Co., Madison, Wisconsin, USA) diluted in 0.1 M TBST, with 5% Skim Milk diluted at 1:3000. The secondary Ab was visualized with NBT/BCIP Tablets (Roche Diagnostics GmbH, Mannheim, Germany) diluted in double distilled water.

### 4.4. Whole-Mount Immunohistochemistry

The embryos and larvae were fixed with 4% paraformaldehyde diluted in filtered seawater for 15 min, dehydrated in increasing concentrations of ethanol diluted in double distilled water, and stored in 70% ethanol at 4 °C until use. The samples were hydrated in decreasing concentrations of ethanol and transferred to 0.1 M phosphate buffered saline with 1% (*v/v*) Tween-20 (PBST). The primary and the secondary antibodies used in this study are listed in Table 1 and Table 2, along with the conditions applied. Finally, the nuclei were counterstained for 5 min with 1–2 μg/mL 4′, 6-diamidino-2-phenylindole (DAPI) diluted in 0.1 M PBST. The samples were examined under a TCS SP8 confocal laser scanning microscope (Leica Microsystems, Co. Japan, Tokyo, Japan). Some of the confocal images were reconstructed three-dimensionally with the following 3-D visualization and analysis software: Avizo software ver.6.1.1 (FEI Visualization Sciences Group, Bordeaux, France), an Amira software (FEI Visualization Sciences Group, Burlington, MA 01803, USA), or ImageJ 1.45 s (National Institutes of Health, Betheda, MD, USA).

### 4.5. FL-Htt-MO Application

Prior to applying *FL-Htt-MO*, 5′-TATTGCCTTTGAGATAAATCTTCAT-carboxyfluorescein-3′ was diluted in Endo-Porter PEG (Gene Tools, LLC. Philomath, OR 97370, USA) after removing the fertilization envelope to ensure access to the cells, as follows. Fresh eggs were obtained by injecting 0.5 M KCl into the coelomic cavity, then were pretreated with 1 mmol/L 3-Amino-1,2,4-triazole (Tokyo Chemical Industry Co., Ltd. Tokyo, Japan) for 10 min, and inseminated [51]. Then, the softened fertilization envelopes were removed by passing the eggs through a 62 µm nylon mesh (Kyoshin Ricoh Inc., Tokyo, Japan). The denuded fertilized eggs were incubated in a mixture of 10 or 20 µM *FL-Htt-MO* [2 µL Stock sol + 4 µL Endo-Porter-PEG in 1 mL FSW (Gene Tools, LLC, https://www.gene-tools.com/content/getting-morpholinos-cultured-cells) (Access on: 3 March 2021)] or in normal FSW in a 24-well plate at a concentration of about 10 eggs/2 mL in a well at 18 °C until 4aPL. At the sBL, 2aPL, and 4aPL stages, they were used for examining ciliary beating activity as will be described below. Aliquots of these 4aPLs were fixed for whole-mount immunohistochemical analysis, as described above.

### 4.6. Cell Proliferation Analysis Using BrdU Incorporation

The 13-hpf mBL were incubated with or without 20 µM *Hp-Htt-MO* at 18 °C. At the 27-hpf early 4aPL stage, 2 mM of BrdU (Sigma Chemical Co. St Louis, MO, USA) was added and incubated for 3 h in 2 mL FSW at 20 larvae/well, in a 24-well plate. Then, they were fixed with 4% paraformaldehyde in FSW for 1 h, washed three times for 10 min each in 0.1 M PBST, incubated for 2 h in 2N HCl, blocked in 5% BSA in 0.1 M PBST for 1 h, and incubated overnight in mouse anti-BrdU Ab (diluted 1:250 in 0.1 M PBST). After the Ab treatment, the samples were further washed three times for 10 min each with 0.1 M PBST, and incubated with Zenon Alexa Fluor 488-tagged anti-rabbit IgG (diluted at 1:300 in 0.1 M PBST) overnight. Then, they were incubated with rabbit anti-Hp-Htt Ab, and then with the secondary Ab of Alexa Fluor 594-tagged anti-rabbit IgG, counter stained with DAPI, and examined under a confocal microscope, as described above.

### 4.7. Swirling Track Pattern Analysis

To observe the swirling track patterns created by the ciliary beating of the embryos and the larvae under the influence of 20 µM *Hp-Htt-MO*, they were placed on hole-slide glass and mixed with marine algae, *Chaetoceros gracilis* (Nisshin Marine Tech. Ltd., Yokohama, Japan), as a marker of the swirling, and then dark-field microscopy was taken using a Canon EOS Kiss X3 camera with darkfield for 3.2s of exposure time [19]. As a negative control, aliquots of embryos and larvae were incubated with 6 µM PEG or in plain FSW, and their swirling patterns were recorded as stated above. The images were converted to black and white images and the contrast was enhanced to visualize the swirls with ImageJ 1.52 (NIH, Bethesda, MD, USA).

Initial examination of the swirling number was carried out using 4aPL alone. We used 35 larvae in plain FSW, 43 larvae in the mixture of 20 µM *Hp-Htt-MO* and 6 µM PEG, and 18 larvae in 6 µM PEG alone, respectively. The swirls created by sBL, 2aPL, and 4aPL were examined using 16 sBL, 12 2aPL, and 59 4aPL for the control FSW and 15 sBL, 13 2aPL, and 39 4aPL for the *Hp-Htt-MO* treatment.

## Figures and Tables

**Table 1:** The list of primary antibodies used in this study.

Antigen	Host	Antibody Type	Applied Dilution	References
Sp-Htt (Hp-Htt)	Rabbit	Polyclonal	1:500	This study
Hp-GAD	Rabbit	Polyclonal	1:500	[23]
Hp-Tjp1	Rabbit	Polyclonal	1:1000	[18]
Hp-5HThpr	Mouse	Polyclonal	1:200	[19]
P4	Mouse	Monoclonal	No dilution	[49]
Serotonin	Rabbit	Polyclonal	1:1000	Sigma-Aldrich, St. Louse, MO, USA
BrdU	Mouse	Polyclonal	1:250	Sigma-Aldrich, St. Louse, MO, USA
Epith-2	Mouse	Monoclonal	1:100	[50]

**Table 2:** The list of secondary antibodies used in this study. All from Invitrogen.

Antigen	Labeled Fluorescent Dye	Applied Dilution
Rabbit	Alexa Fluor 488	1:500
Rabbit	Alexa Fluor 568	1:500
Rabbit	Alexa Fluor 594	1:500
Rabbit	Zenon Alexa Fluor 488	1:300
Rabbit	Zenon Alexa Fluor 568	1:300
Rabbit	Zenon Alexa Fluor 594	1:300
Mouse	Zenon Alexa Fluor 488	1:300
Mouse	Zenon Alexa Fluor 568	1:300

## Data Availability

Data is contained within the article or supplementary material.

## Supplementary Materials

Supplementary Materials can be found at https://www.mdpi.com/article/10.3390/ijms22105116/s1.

## Abbreviations

CBAS	Ciliary band-associated strand
*FL-Htt-MO*	*Fluorescein-tagged Hp-Htt morpholino*
GAD	Glutamic acid decarboxylase
HTT	Huntingtin (Huntington’s disease protein)
Hp-Htt	Huntington’s disease protein of the sea urchin *Hemicentrotus pulcherrimus*
l-, r-ALA	Left- and right-anterolateral arm
l-, r-POA	Left- and right-postoral arm
PEG	Endo-Porter-Polyethylene glycol
Tjp1	Tight junction protein 1
2aPL	Two-arm pluteus larva
4aPL	Four-arm pluteus larva
5HT	Serotonin
5HThpr	Serotonin receptor
